# Estimation of Tissue Attenuation from Ultrasonic B-Mode Images—Spectral-Log-Difference and Method-of-Moments Algorithms Compared †

**DOI:** 10.3390/s21072548

**Published:** 2021-04-05

**Authors:** Dinah Maria Brandner, Xiran Cai, Josquin Foiret, Katherine W. Ferrara, Bernhard G. Zagar

**Affiliations:** 1 Institute for Measurement Technology, Johannes Kepler University Linz, 4040 Linz, Austria; bernhard.zagar@jku.at; 2 Linz Center of Mechatronics Ltd. (LCM), 4040 Linz, Austria; 3 Department of Radiology, Stanford University, Palo Alto, CA 94304, USA; caixr@shanghaitech.edu.cn (X.C.); jlfoiret@stanford.edu (J.F.); kwferrar@stanford.edu (K.W.F.)

**Keywords:** ultrasound imaging, attenuation imaging, k-Wave, ultrasound simulation, spectral log difference technique, method of moments, tissue mimicking materials

## Abstract

We report on results from the comparison of two algorithms designed to estimate the attenuation coefficient from ultrasonic B-mode scans obtained from a numerical phantom simulating an ultrasound breast scan. It is well documented that this parameter significantly diverges between normal tissue and malignant lesions. To improve the diagnostic accuracy it is of great importance to devise and test algorithms that facilitate the accurate, low variance and spatially resolved estimation of the tissue’s attenuation properties. A numerical phantom is realized using *k*-Wave, which is an open source Matlab toolbox for the time-domain simulation of acoustic wave fields that facilitates both linear and nonlinear wave propagation in homogeneous and heterogeneous tissue, as compared to strictly linear ultrasound simulation tools like Field II. *k*-Wave allows to simulate arbitrary distributions, resolved down to single voxel sizes, of parameters including the speed of sound, mass density, scattering strength and to include power law acoustic absorption necessary for simulation tasks in medical diagnostic ultrasound. We analyze the properties and the attainable accuracy of both the spectral-log-difference technique, and a statistical moments based approach and compare the results to known reference values from the sound field simulation.

## 1. Introduction

Quantitative ultrasound (QUS) seeks to gather more information derived from B-mode images than is attainable by displaying just the amplitudes of the complex envelope of the time gain compensated and log-compressed echo signals, which is the information typically conveyed by B-mode images. For quantitative ultrasound total attenuation as well as backscatter coefficient have been shown to provide additional diagnostic information [1,2,3]. A good overview on the benefits of QUS in the diagnosis of malignant processes as well as a discussion of the applied methods is given, e.g., in [4,5]. Attenuation maps can show contrast between regions that do not appear differently in a standard B-mode image. Most of the methods in QUS are based on the estimation of frequency domain parameters, like the spectral-log-difference method that uses the difference in the spectral magnitude at different depths to estimate the locally averaged attenuation from ultrasonic backscatter data, or the method-of-moments, which also takes into account the downshift of the spectrum caused by the frequency dependent attenuation of the propagating wave. Those methods all rely on the assumption, which in most cases is not valid, that the backscattering coefficient does not change with the considered region of interest (ROI). This fact requires to apply a rather large spectral analysis window which limits the attainable spatial resolution. Superficial tumors like those typical for the breast or prostate are particularly well suited to be characterized via QUS, thus we decided to work and report on results of malignancies of the human breast.

Table 1 lists the acousto-mechanical properties of various benign and malignant breast tissue types and one can observe that benign tissue types like glandular tissue, fat, or supportive tissue exhibit attenuation coefficients $\alpha_{0}$ close to and around $\alpha_{0}$ = 1.0 dB/(MHz cm), whereas benign cysts are closer to $\alpha_{0}$ = 0.2 dB/(MHz cm) and malignant masses show values of around $\alpha_{0}$ = 2.0 dB/(MHz cm) [4,6,7]. From those numerical values one might conclude that being able to devise an unbiased estimator for the attenuation coefficient that has a standard deviation for $\alpha_{0}\leq 0.3$ would give a diagnostic benefit. Although there still does not exist a robust estimation method to determine spatially resolved attenuation coefficient, several authors have addressed that problem and developed a variety of methods both in the time as well as in the frequency domain [1,2,3]. We employ a sound field simulation software (*k*-Wave [8]) to generate B-mode scans of a numerically very well characterized volume and are thus able to compare the estimation results obtained for two techniques, the spectral-log-difference technique [9] and the method of moments [10,11].

The paper is organized as follows. Section 2.1 gives an introduction to the production and characterization of tissue mimicking phantoms that can be isonified and analyzed by an ultrasound scanner to provide real B-mode ultrasonic scans the algorithms can be trained on and characterized. Section 2.2 discusses the attenuation ultrasonic waves experience while propagating into the tissue, being scattered, diffracted, and refracted by some inhomogeneities and propagating back to the receiving aperture of the ultrasonic probe. Section 2.3 discusses properties of the ultrasound simulation software considered in this work and Section 2.4 discusses the two algorithms for estimating tissue attenuation from B-mode scan data that were analyzed for their statistical properties. Finally Section 3 presents and discusses numerical results and B-mode images augmented by color-coded attenuation data. Section 4 gives a discussion and an outlook on further research to be done.

## 2. Methods

In this section’s first part we detail the production and characterization of tissue mimicking phantoms, that still provide a gold standard for evaluation and comparison of digital signal processing methods to estimate various tissue properties. These phantoms allow for small systematic errors, a good reproducibility and small variances of mimicked tissue parameters.

We continue on with a discussion of the physics on ultrasonic attenuation governed by absorption and scattering properties of the isonified medium, be it a phantom or human tissue. We then continue with a discussion and the well-founded selection of a highly sophisticated ultrasound simulation environment (*k*-Wave, ref. [13]), which allows to simulate the full-blown, non-linear wave equation for an absorbing and structured medium, and report on results obtained and their numerical validity even for highly absorbing media as typically seen in malignant processes of human tissue.

The subsequent section details the mathematical basis for two estimators and we report on results obtained for a non-structured but scattering and absorbing numerical phantom and show that both estimators are able to provide low variance estimates of tissue attenuation.

### 2.1. Tissue-Mimicking Phantoms

With tissue-mimicking materials it is possible to produce phantoms which mimic human breast tissue in all acoustic aspects [14,15]. Therefore we focussed on finding a connection between a material composition and the resulting acoustic properties, providing the opportunity to later test the estimation algorithms.

The essential ingredients for the production of tissue-mimicking phantoms are water, agar for the solidification and silicon carbide for the scattering effect, which leads to the grainy pattern, the so-called speckle exhibited in ultrasound images obtained by clinical equipment. To prevent the emergence of microbubbles that would lead to the distortion of the measurements of the phantoms’ acoustic properties, it is important to use distilled and degassed water. There are additional components to the mixture, which have different effects on the phantom’s acoustic properties, glycerol for instance increases the speed of sound, aluminium oxide of different grain sizes on the other hand increases the attenuation coefficient [14].

As we aim to estimate attenuation coefficients, we used the list of composition of the International Electrotechnical Commission (IEC) TMM [16], see Table 2, and varied the concentration of aluminium oxide (grain sizes 0.3 and 3 μm) to determine the effect on the acoustic properties of the phantoms.

**Figure 1:**
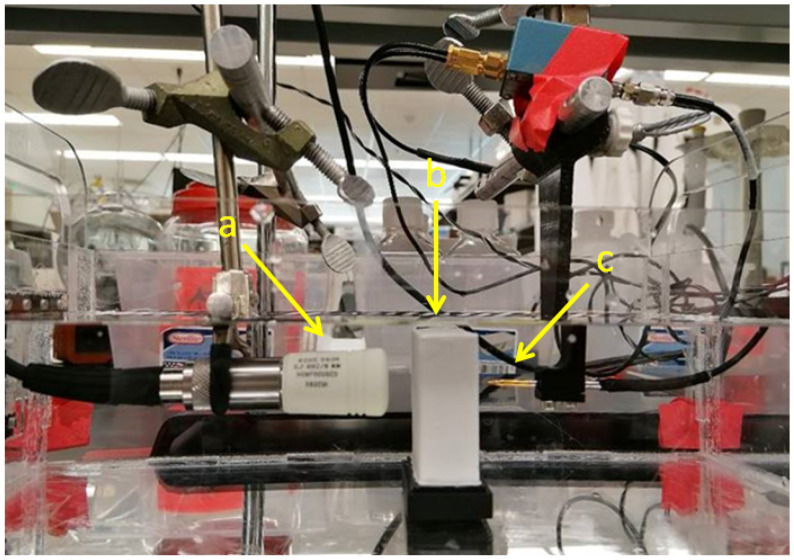
Setup used for the measurement of tissue-mimicking phantoms. (**a**) Piston-type US transducer (ROHE-5604), (**b**) phantom, (**c**) needle hydrophone (HNA-0400, ONDA, Sunnyvale, CA, USA) with amplifier (AH-1100, ONDA, Sunnyvale, CA, USA). The thickness of the phantom is 2 cm.

For measurements an US setup as shown in Figure 1 is used in transmission mode. After a pulse generator (here: 5072PR, Olympus Corporation, Shinjuku, Tokyo, Japan) excites the US transducer and a needle hydrophone measures the sound pressure level with and without the phantom in place, the attenuation for a given thickness can then be directly determined using the spectral-log-difference technique (SLD), which will be discussed in more detail below [9]. A short pulse in the time domain is desired as it results in a high spatial resolution in imaging applications. The transducer was driven with a narrow pulse exciting its $f_{c}=5.0$ MHz center frequency, large relative bandwidth, resulting in a single cycle pressure wave emitted into the volume. According to the SLD method, the attenuation coefficient is calculated from the log-difference between the two spectra,

(1) $$\alpha \left(f\right)=-\frac{20}{d}\log_{10}\frac{S_{ph}\left(f\right)}{S_{ref}\left(f\right)},$$

where *d* is the thickness of the phantom and $S_{ph}\left(f\right)$ and $S_{ref}\left(f\right)$ are the magnitude spectra of the phantom data and the reference data. The reference data were acquired with the same settings as were used without the phantom in place.

The setup (Figure 1) was used to estimate the attenuation coefficient $\alpha_{0}$ for different aluminium oxide concentrations in tissue mimicking phantoms.

**Figure 2:**
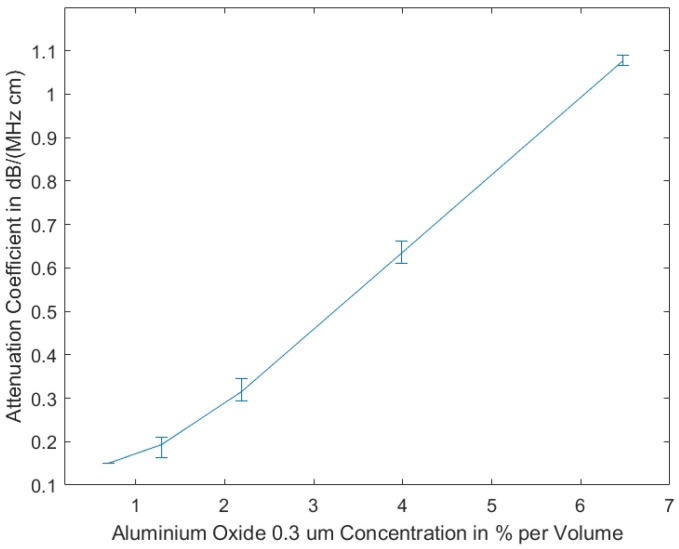
Attenuation coefficient vs. concentration of aluminum oxide (grain size 0.3 μm) for homogeneously prepared tissue mimicking phantoms (as shown in Figure 1b). Reprinted from ref. [15].

Our studies show that the measurements of the attenuation coefficients of the tissue-mimicking phantoms produced using 0.3 μm aluminium oxide show less variance, due to homogeneity resulting from smaller grain size, as compared to the measurements of the phantoms including aluminium oxide 3 μm. In Figure 2, the measurement results are displayed, showing that the attenuation coefficient is almost linearly dependent on the volume concentration of aluminium oxide.

### 2.2. Ultrasound Attenuation

When an ultrasound wave propagates, it generally loses some acoustic energy to random thermal motion resulting in acoustic absorption. Scattering loss, as a second component of attenuation, is caused by the partial interception by the receive aperture of the angular distribution of the backscattered signal of sub-wavelength sized scatterers. Scattering can be classified depending on the relative size of the scattering inhomogenities of the tissue relative to the interrogating wavelength λ=c/f, with *c*, being the speed of sound, and *f*, the excitation frequency. Living cells are sub-wavelength scatterers with concentrations of more than 25 per resolution cell ([4], Chapter 9) and act diffusive. Larger inhomogeneities of length scales on the order of λ with concentrations lower than one per resolution cell are independent and distinguishable by their characteristics.

Absorption and scattering combined effects are summarized as attenuation and can be modeled as a sound-absorbing fluid in which the absorption follows a frequency power law of the form

(2) $$\alpha^{\prime }=\alpha_{0}^{\prime }\;\omega^{y}\;\;\;\mathrm{or}\;\;\;\alpha =\alpha_{0}\;f^{y}\;,$$

where, $\alpha^{\prime }$, is the absorption coefficient in coherent units of Np/m, $\alpha_{0}^{\prime }$, is the power law prefactor in Np (rad/s)${}^{-y}$ m${}^{-1}$, *y* is the power law exponent, ω, is the angular frequency and the resulting attenuation is measured in the coherent units Np (rad/s)${}^{-y}$ m${}^{-1}$.

In medical terms, the attenuation will be referred to the frequency *f* in Hz, and α be given in dB/cm as opposed to Np/m, thus further on we will be using $\alpha_{0}$ in dB/(MHz${}^{y}$ cm), and α in dB/cm. The power law exponent, *y*, for human tissue is close to unity, as can be seen from Table 1 [4], so the SLD algorithm will assume y=1.00, whereas the also discussed method-of-moments (MoM) will consider *y* as variable to also be estimated from the B-mode scan lines. In ultrasonic imaging of human tissue, the propagating wave cannot be observed directly but its amplitude needs to be estimated from observations of the backscattered signals as seen at the location of the ultrasound transducer’s receiving aperture. The estimation of the spatially varying attenuation coefficient α=α(depth) is thus an ill-posed inverse problem that needs to be solved.

The backscattering occurs due to the cell-based structure of all biological tissues (except maybe fluid filled cysts that appear to be almost anechoic and thus image dark in B-mode scans). These scattering centers are very small sized as compared to the US wavelength λ, thus scatter very weakly but are present at a high scatterer number density randomly distributed in space. So the overall action of these scatterers can be modeled in a first order approximation as a spatial white noise process, which, assuming an almost constant speed of sound *c*, gives an impulse response-like behavior to the imaging process. How strongly valid this simplifying assumption is, needs to be thoroughly analyzed, but this is out of scope of this contribution.

In this paper, we concentrate on the estimation of the attenuation parameter α (in units of dB/cm). US absorption, as characterized by Equation (2), for soft biological tissues ranges from $\alpha_{0}=0.03$ dB/(MHz cm) for water up to a maximum $\alpha_{0}=4.0$ dB/(MHz cm) for certain lesions. Some authors have directed their interest into estimating the backscatter-coefficient, too, in order to then be able to estimate the attenuation coefficient much more accurately [17].

Testing of algorithms for estimating absorption parameters from acquired B-mode images, an example of which is shown in Figure 5, right, requires a well characterized set of training data that can be obtained by either tissue mimicking phantoms (see Section 2.1), exhibiting fairly homogeneous properties that can both be directly measured using the transmission mode or the backscatter mode as in imaging US, respectively.

Another less involved way to obtain calibration data for testing α–estimators is using simulation software to solve the US wave equation directly for known properties of the propagation medium, including source terms, absorption and dispersion relations and potentially also the nonlinear pressure-density relation. An open source time-domain simulation software package developed for this purpose is *k*-Wave [8,13] which will be discussed next.

### 2.3. Discussion of Simulation Software

There are several open source software packages available that allow the time-domain simulation of propagating acoustic waves including various aspects of an ultrasonic imaging system. A good overview is given in [18]. We evaluated two software tools, Field II [19] and *k*-Wave [8,13] for their suitability to the problem of estimating tissue properties (attenuation coefficient in particular) besides simulating the imaging capabilities of an ultrasonic scanner. Field II being orders of magnitude faster in execution than the alternative and finally chosen *k*-Wave software, soon turned out to be unsuitable, since it relies on the spatial impulse response method [20] that only allows one to simulate linear wave propagation, a restriction we could not accept since the simulation results should, in as many aspects as possible, mimic the real and physical propagation of sound waves in a medium (breast tissue).

The much more powerful *k*-Wave simulation environment, on the other hand, is able to select all possible parameters of the propagation medium on a voxel by voxel basis and simulates linear and non-linear wave propagation for an arbitrary distribution of heterogeneous material parameters and power law acoustic absorption although at the cost of a dramatic increase in necessary processing power.

If a reasonably sized, structured volume is to be simulated, e.g., 30×30×10 mm${}^{3}$ at a spatial resolution of 30 μm as demanded for an f=5.0 MHz excitation frequency, a data array size of 333 Mvoxels results and needs to be simulated at time increments of a few ns up over a total simulation time, allowing the wave to propagate back and forth along the longest geometrical path in the volume. This results for the example in about 10,000 time increments @ 5 ns to simulate a single B-mode line. This size can only be handled utilizing either a supercomputing environment or employing state of the art graphics processing units (GPU) with several thousand GPU cores programmed to operate concurrently.

For a numerically stable solution, of the non-linear partial differential equations governing the wave propagation at least 10 grid points per acoustic wavelength λ are generally required. The problem is confounded further by the requirement for small time steps to keep the simulation numerically stable and to minimize unwanted numerical errors to accumulate.

We tested the suitability of an NVIDIA Titan RTX 24 GB GPU (Nvidia, Santa Clara, CA, USA) that is marginally able to simulate such a huge volume array in an acceptable simulation time frame of a few days (for 201 B-mode scan lines).

Obtaining numerical stability and keeping the round-off errors small, an IEEE-854 double precision number format should be selected. GPUs, however, typically are restricted to single-precision arithmetic and, what is not widely known, do not realize the full IEEE-754 single precision format, that would provide 23 bits for the mantissa (=$2^{-23}\approx 1.19$· 10${}^{-7}$ in relative resolution resulting in a dynamic range for the sound pressure of approximately 130 dB) but specify for some basic operations just 21 or even only 20 significant bits [21].

**Figure 3:**
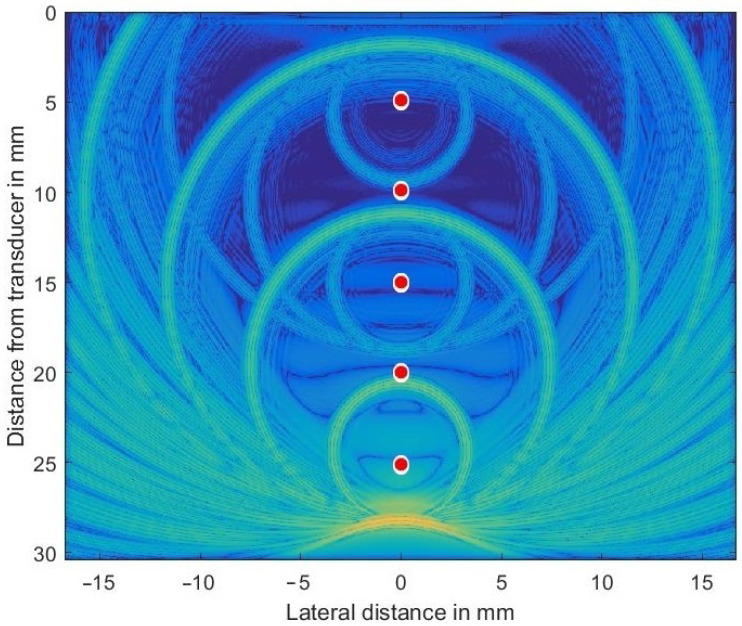
Ultrasonic wave’s envelope propagating downwards, focused at 35 mm and being scattered off discretely placed scatterers (indicated as red circles in exaggerated size). The chosen colormap for the log-compressed display extends from 100 kPa (orange) to 1 Pa (in blue).

In order to check the validity of the simulation in *k*-Wave, a homogeneous and isotropic medium was simulated that included five discretely placed scatterers (at depths of d={5,10,15,20,25} mm) and $\alpha_{0}$ was set at 2.3 dB/(MHz cm) throughout the volume to generate both large and very small sound pressure levels propagating concurrently through the volume. The transducer (center frequency f=5.0 MHz) was focused both in azimuth and elevation at 35 mm in depth. For such a simple problem an analytic solution can be given and so allows the validity of the numerical results to be checked. Figure 3 shows the complex envelope of the propagating wave some 17.5 μs after simulation started. The main pulse located at 28 mm depth had excited all 5 scatterers while propagating downwards.

The secondary waves emitted from scatterers located at 10, 15, 20, and 25 mm depths are still clearly visible (the secondary wave from scatterer at 5 mm has already left the simulated volume), as are all re-scattered waves. Very low spatial frequency waves, as seen in the very center, are an indication of numerical problems encountered if the very large dynamic range necessary cannot be met by the GPU arithmetic.

**Figure 4:**
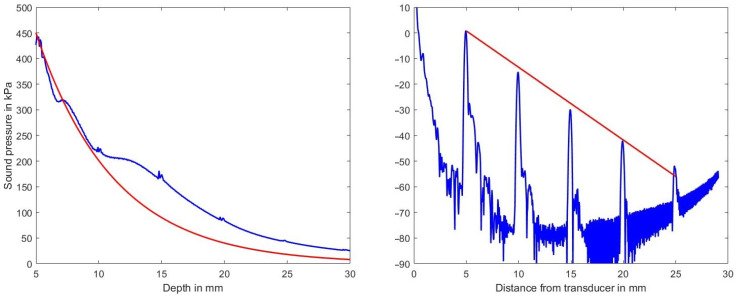
Left diagram: decrease of sound pressure amplitude vs. depth along the center line within the volume in blue and the expected decrease in sound pressure for the assumed attenuation coefficient of 2.3 dB/(MHz cm) in red. Right diagram decrease of sound pressure level vs. depth estimated from a B-mode line (in dB ref. 100 kPa). Indicated in red is the expected decrease in amplitude vs. depth for the chosen attenuation of 2.3 dB/(MHz cm).

Figure 4, left, shows the sound pressure amplitude vs. depth in blue on a linear scale and the expected decrease in amplitude (according to the selected medium parameters of 2.3 dB/(MHz cm)) in red. The deviation between the two graphs is attributed to the focusing action of the phased-array transducer which is focusing at a depth of 35 mm.

Clearly visible also is the effect of the scatterers at d={5,10,15,20,25} mm. Figure 4, right, on the other hand, shows on a logarithmic scale in blue the sound pressure level (in dB ref. 100 kPa) and in red the expected decrease due to the chosen attenuation coefficient as derived from a single B-mode line. Simulation artifacts at low sound pressure values increase the noise floor beyond 17 mm in depth and those artifacts raise the noise floor at greater depths.

Concluding this section, *k*-Wave run on a GPU gives numerical stable results for attenuation coefficients up to and including 2.5 dB/(MHz cm) for a typical volume size, which is sufficient for simulating most breast tissue types.

### 2.4. Estimators of Tissue Attenuation

Of all fluid properties, quantities that describe loss are among the most challenging to estimate. In part, this is due to superimposed dissipative mechanisms, as described above, and diffraction effects from sub-wavelength scatterers. Inherent to these phenomena is a specific frequency dependence as given by Equation (2). According to Table 1 the power law exponent *y* varies with the respective absorption mechanism. Liquid-filled cysts behave similar to like pure water and thus have an exponent close to y=2.0, combined with an attenuation of $\alpha_{0}=0.2$ dB/(MHz cm), whereas solid masses have an exponent closer to y=1.0 combined with $\alpha_{0}$ of 2.50 dB/(MHz cm). In order to improve the diagnostic accuracy, both parameters *y* and $\alpha_{0}$ are of interest and shall be estimated.

Several authors have elaborated on the estimation of α, or $\alpha_{0}$, respectively, and *y* but conclude that it is an ill-posed mathematical problem [4,9]. This difficulty arises in part from the demand to spatially map these parameters in order to detect small to very small lesions. The severe tradeoff between estimator’s variance and spatial resolution impacts both methods analyzed in this work.

**Figure 5:**
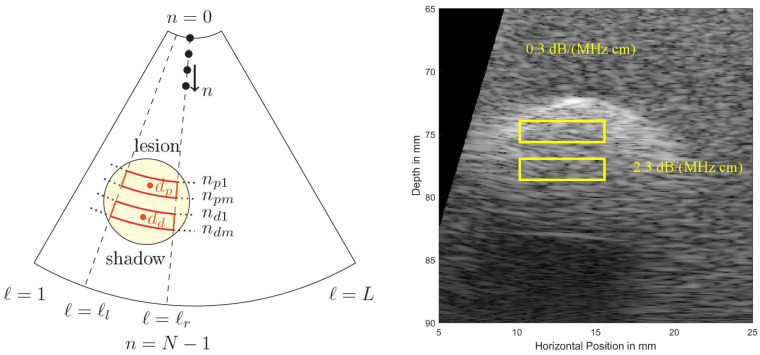
(**Left**): To define locations and indices within a sector scan used in the estimation algorithms. At the top the US transducer is assumed operating as a phased array. (**Right**): Simulated US B-mode image, exhibiting typical US speckles and shadowing behind a strongly absorbing region (a simulated lesion). Distal (bottom, subscript (...)${}_{d}$) and proximal (top, subscript (...)${}_{p}$) windows are indicated.

Figure 5 shows a cropped B-mode scan exhibiting the typical US speckles that are detrimental in the estimation process. Furthermore, the distal and proximal data windows over which both estimation algorithms operate are indicated. Note that here a phased array transducer was used with a steering angle of $\pm 15^{\circ }$. The sketch in Figure 5, left, defines all indices used in both algorithms. Index 1≤ℓ≤L is indicating the lateral sector scan line (typically going up to and beyond L=200), and index 0≤n<N−1 is indicating the depth dimension (typically being sampled at and beyond an $f_{s}=200$ MHz sampling rate). Given that the speed of sound, c≈1540 m/s, on the order of $N=10^{4}$ samples are acquired for each scan line for a typical ≈5 cm depth range.

In our work, we compare results obtained by the spectral-log-difference (SLD) method [3,9,22] and the statistical moments (MoM) based approach [10,11] for three numerical phantoms.

The first phantom is a homogeneous medium with $\alpha_{0}=1.00$ dB/(MHz cm) used to obtain statistical properties of the estimators. The second and third phantoms mimic a liquid filled cyst with lower than average attenuation but a power-law exponent of y=1.2, and a dense lesion with $\alpha_{0}=2.5$ dB/(MHz cm) embedded in fat as medium. The SLD allows to estimate the attenuation coefficient only, the MoM also allows to estimate higher order terms of the frequency dependency of α that might help to differentiate between normal tissue (y≈1.0), and fluid filled cysts (y>1.0).

#### 2.4.1. Spectral-Log-Difference Method

In order to estimate $\alpha_{0}$ over a region of interest (ROI) set by range-gated proximal and distal windows within a B-mode scan of a breast, the same time-gain-compensation (TGC), and the same power settings are used to obtain backscattered signals from different depths over a lateral range of B-mode scan lines $\ell_{l}\leq \ell <\ell_{r}$ (see Figure 5) to then be averaged. The authors in [23] report results using block sizes of 22.5·λ axially by 43·λ laterally so considering an f=5.0 MHz transducer this results in a block size of 6.93×13 mm${}^{2}$.

We define $S_{w}\left(f_{k}\right)$, the power spectral density of the appropriately time-windowed (subscript ${\left(...\right)}_{w}$) sequence $s_{w}\left(n\right)$ evaluated at frequencies $f_{s}\cdot k/M;\;0\leq k\leq \left(M-1\right)$, with *k*, the frequency index, $f_{s}$ the sampling frequency in Hz, and $M=\left(n_{pm}-n_{p1}+1\right)/2=\left(n_{dm}-n_{d1}+1\right)/2$, the lengths of the time windowed sequences. Please note, this window or tapering function used to suppress spectral side lobes is to be distinguished from the above mentioned depth windows that allow to select spectra from different depths.

$$\begin{array}{cc} S_{w}\left(f_{k}\right) & =S_{w}\left(\frac{f_{s}\cdot k}{M}\right)=\frac{M}{2\pi f_{s}}\cdot {\left|F\left\{s_{w}\left(m\right)\right\}\right|}^{2}=\\ \; & =\frac{M}{2\pi f_{s}}{\left|\frac{1}{M}\cdot \sum_{m=0}^{M-1}s_{w}\left(m\right)\cdot e^{-j\cdot \frac{2\pi }{M}mk}\right|}^{2} \end{array}$$

Now define B(ℓ,n) the transducer’s output signal representing the TGC-compensated B-mode image with *ℓ* being the scan line index of the sector scan, and *n* being the time index representing the depth dimension. Depth $d\left(n\right)=n\cdot c/\left(2f_{s}\right)$ (assuming an approximately constant over depth speed of sound *c*).

Both windows are limited laterally by $\ell_{l}\leq \ell \leq \ell_{r}$, thus in total $L_{e}=\ell_{r}-\ell_{l}+1$ scan lines are averaged over (denoted by 〈⋯〉) in the following spectral estimation process. The proximal window extends between $n_{p1}\leq n\leq n_{pm}$, and the distal window between $n_{d1}\leq n\leq n_{dm}$ samples.

Using the above definitions, the power spectral density of a depth-windowed region (mean depth for the proximal window $d_{p}$, and for the distal window $d_{d}$) in a statistically homogeneous tissue, varying only in the attenuation coefficient, and averaged over all $L_{e}=\ell_{r}-\ell_{l}+1$ selected scan lines is given by (subscripts *p* and *d* indicate the proximal and distal ROIs).

$$\langle S_{p}\left(f,d_{p}\right)\rangle =P\left(f\right)D\left(f\right)A\left(f,d\right)B\left(f,d\right)e^{-4\alpha \left(f\right)d_{p}}$$

for the proximal window, and

$$\langle S_{d}\left(f,d_{d}\right)\rangle =P\left(f\right)D\left(f\right)A\left(f,d\right)B\left(f,d\right)e^{-4\alpha \left(f\right)d_{d}}$$

for the distal window, where P(f) represents the combined effect of the transmit pulse and the sensitivity (electro-acoustic and acousto-electric transfer functions) of the transducer, D(f)=D(f,d) denotes the effects of focusing and diffraction that are related to the transducer geometry and its numerical aperture NA at average depth $d=(d_{p}+d_{d})/2$, A(f)=A(f,d) is the cumulative attenuation along the propagation path from the surface of the transducer to depth *d* of the respective depth window which corresponds to the centers $d_{p}$ and $d_{d}$, respectively, of the ROIs, α(f) is the attenuation coefficient within the considered ROIs, B(f)=B(f,d) is a result of the scattering properties of the tissue within the ROIs, namely the effective scatterer size, the scatterer number density, and the mean square variation in acoustic impedance between the scatterers and the background.

Dividing the power spectra obtained from different average depths $d_{p}$ and $d_{d}$ and assuming that the tissue within these depth ranges is homogeneous and isotropic, i.e., the scattering term B(f) does not vary with depth, yields (for depths parameters in cm):

$$Y\left(f\right)=\frac{S_{p}\left(f\right)}{S_{d}\left(f\right)}=\frac{A\left(f,d_{p}\right)B\left(f,d_{p}\right)}{A\left(f,d_{d}\right)B\left(f,d_{d}\right)}\cdot e^{4\left(d_{d}-d_{p}\right)\alpha \left(f\right)}\;.$$

Taking the logarithm (to the base 10 to express the spectra in dB/Hz, and thus the attenuation coefficient in dB/(MHz cm)) yields:

$$\begin{array}{cc} \log\left(Y(f)\right) & =\frac{1}{20}\ln\left(10\right)\cdot 4\left(d_{d}-d_{p}\right)\alpha \left(f\right)\cdot \\ \; & \cdot \frac{A\left(f,d_{p}\right)B\left(f,d_{p}\right)}{A\left(f,d_{d}\right)B\left(f,d_{d}\right)}\;,\\ \; & \approx \frac{1}{20}\ln\left(10\right)\cdot 4\left(d_{d}-d_{p}\right)\alpha \left(f\right)\\ \; & =\frac{1}{10}\ln\left(10\right)\cdot 2\left(d_{d}-d_{p}\right)\alpha \left(f\right)\;. \end{array}$$

In Equation (3) [24] the factor 4 stems from the fact that the attenuation ratio as measured in Np is referring to the attenuation of field quantities (as opposed to dB which gauges power quantities) combined with the round-trip propagation that effectively doubles the associated path lengths. The attenuation coefficient of the sample (in dB/(MHz cm)) can be estimated at each frequency component $f_{k}$ (see Equation (3)) by calculating γ, the slope of the straight line that fits the log-ratio of the two spectra Y(f), i.e., the slope of the straight line that fits Equation (3) versus depth. If the attenuation as measured in dB is assumed to increase linearly with frequency, then the attenuation coefficient’s slope is used as a measure for the attenuation in the tissue of interest. The attenuation coefficient can then be simplified to

(3) $$\alpha \left(f\right)=\alpha_{0}\cdot f$$

where the parameter $\alpha_{0}$ is the attenuation coefficient’s slope in dB/(MHz cm) that can be estimated by finding the slope that fits Equation (3).

**Figure 6:**
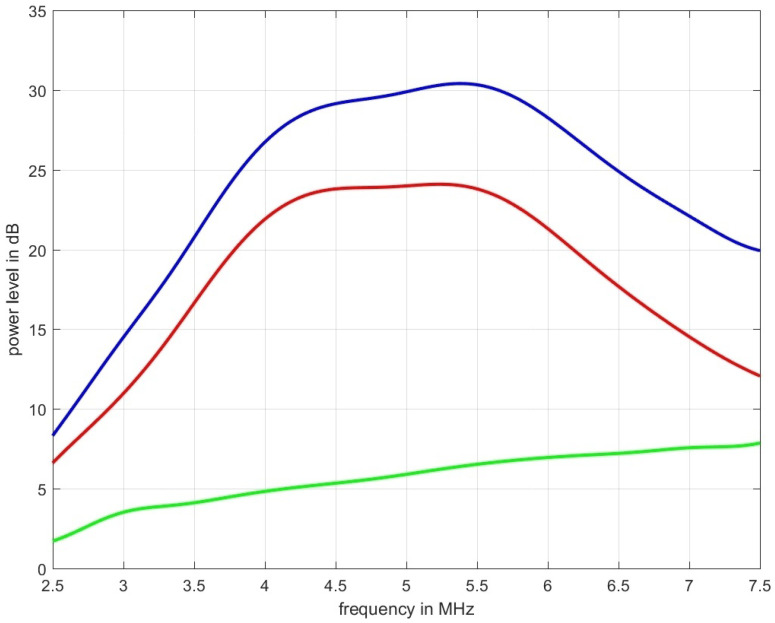
The blue graph represents the power spectral density for the ultrasound propagation in pure water, the red line is the measured spectrum obtained over the thickness of 2 cm of the tissue mimicking phantom depicted in Figure 1 which was designed to result in an $\alpha_{0}$ of 0.6 dB/(MHz cm), and the green line is the spectral-log-difference. The two estimators used, led to an estimated attenuation of: ${\hat{\alpha }}_{0}{|}_{SLD}$ = 0.5663 dB/(MHz cm) and ${\hat{\alpha }}_{0}{|}_{MoM}$ = 0.5759 dB/(MHz cm).

Figure 6 shows measurement data acquired for the tissue mimicking phantom shown in Figure 1. The blue graph represents the power spectral density for the ultrasound propagation in pure water, the red line is the measured spectrum with the phantom inserted, and the green line is the spectral-log-difference to which a straight line could be fitted to obtain $\alpha_{0}$ (in this case ${\hat{\alpha }}_{0}{|}_{SLD}=0.5663$ dB/(MHz cm)).

#### 2.4.2. Method-of-Moments (MoM)

Another method for estimating the attenuation coefficient as discussed in the literature [10,11] is again based on the power spectral densities $S_{p}\left(f\right)$ and $S_{d}\left(f\right)$ averaged over two spatially limited regions of interest (cf. Figure 5) from $\ell_{l}$ to $\ell_{r}$ laterally and from $c\cdot dt\cdot n_{dm}/2$ to $c\cdot dt\cdot n_{d1}/2$ for the distal, and from $c\cdot dt\cdot n_{pm}/2$ to $c\cdot dt\cdot n_{pm}/2$ for the proximal window, respectively, radially.

(4) $$\begin{array}{cc} S_{p}\left(f\right) & =\underset{P\left(f\right)D\left(f\right)A\left(f,d_{p}\right)B\left(f,d_{p}\right)}{\underset{︸}{S_{(}f)}}\cdot e^{-\alpha \left(f\right)\cdot 2\cdot d_{p}}\\ S_{d}\left(f\right) & =\underset{P\left(f\right)D\left(f\right)A\left(f,d_{d}\right)B\left(f,d_{d}\right)}{\underset{︸}{S_{(}f)}}\cdot e^{-\alpha \left(f\right)\cdot 2\cdot d_{d}} \end{array}$$

By taking their zeroth $m_{0,S}\left(d\right)$ to second order $m_{2,S}\left(d\right)$ moments according to

(5) $$\begin{array}{cc} m_{i,S}\left(d\right) & =\int_{0}^{+\infty }f^{i}\cdot S\left(f,d\right)\;df=\int_{0}^{+\infty }f^{i}\cdot S\left(f\right)\cdot e^{-\alpha (f)\cdot 2\cdot d}\;df\;; \end{array}$$

*i* being the moment order, and expanding α(f) into a power series:

(6) $$\alpha \left(f\right)=\sum_{n}a_{n}\cdot f^{n}$$

and noting that the derivative $\partial_{d}m_{i,S}\left(d\right)$ can be written as:

(7) $$\begin{array}{cc} \partial_{d}m_{i,S}\left(d\right) & =\int_{0}^{+\infty }f^{i}\left(-\sum_{n}a^{n}\cdot f^{n}\right)S\left(f\right)\cdot e^{-d\cdot \sum_{n}a_{n}\cdot f^{n}}df=\\ \; & =-\sum_{n}a_{n}\cdot \int_{0}^{+\infty }f^{\left(i+n\right)}\cdot S\left(f\right)\cdot e^{-d\cdot \sum_{n}a_{n}\cdot f^{n}}df=\\ \; & =-\sum_{n}a_{n}\cdot m_{i+n,S}\left(d\right) \end{array}$$

and expressed via the (i+n)th moment of the spectrum S(f).

The parameter $a_{1}$, which is equivalent to $\alpha_{0}\left(f\right)$, and $a_{2}$, which is equivalent to *y* of Equation (2) can then be expressed as:

(8) $$\begin{array}{cc} a_{1}={\hat{\alpha }}_{0}\left(f\right) & =\frac{1}{d_{d}-d_{p}}\cdot \frac{m_{0,S}\left(d_{p}\right)}{m_{1,S}\left(d_{p}\right)}\cdot \ln\left(\frac{m_{0,S}\left(d_{p}\right)}{m_{0,S}\left(d_{d}\right)}\right)\;,\\ a_{2}\to {\hat{a}}_{2} & =\frac{1}{d_{d}-d_{p}}\cdot \frac{m_{0,S}\left(d_{p}\right)}{m_{2,S}\left(d_{p}\right)}\cdot \ln\left(\frac{m_{0,S}\left(d_{p}\right)}{m_{0,S}\left(d_{d}\right)}\right)\;. \end{array}$$

As a note: in estimating the ultrasound’s attenuation the method-of-moments not only utilizes the decline in amplitude with increasing depth *d* but in addition takes into account that the frequency dependent attenuation also reduces the center frequency $f_{c}$ of the propagating wave. It thus should allow for smaller variances of the estimators. Our analysis, however, does not seem to support that assumption, as can be seen by observing the estimation results shown in the following section.

## 3. Results

Both algorithms were tested by analyzing simulated phantoms with parameters listed in Table 3. To guarantee numerical stability and valid results over an extended dynamic range, the *k*-Wave simulation environment, utilizing the so-called pseudospectral method [13], requires a spatial discretization to be reduced to less than λ/10.

A randomly structured macroscopically homogeneous phantom allows to show the unbiasedness of both algorithms even over a large dynamic range of the ultrasound returns. Subsequently a low-attenuation cyst and a high attenuating lesion embedded in this homogeneous medium were simulated and analyzed.

### 3.1. Homogeneous Phantom

**Figure 7:**
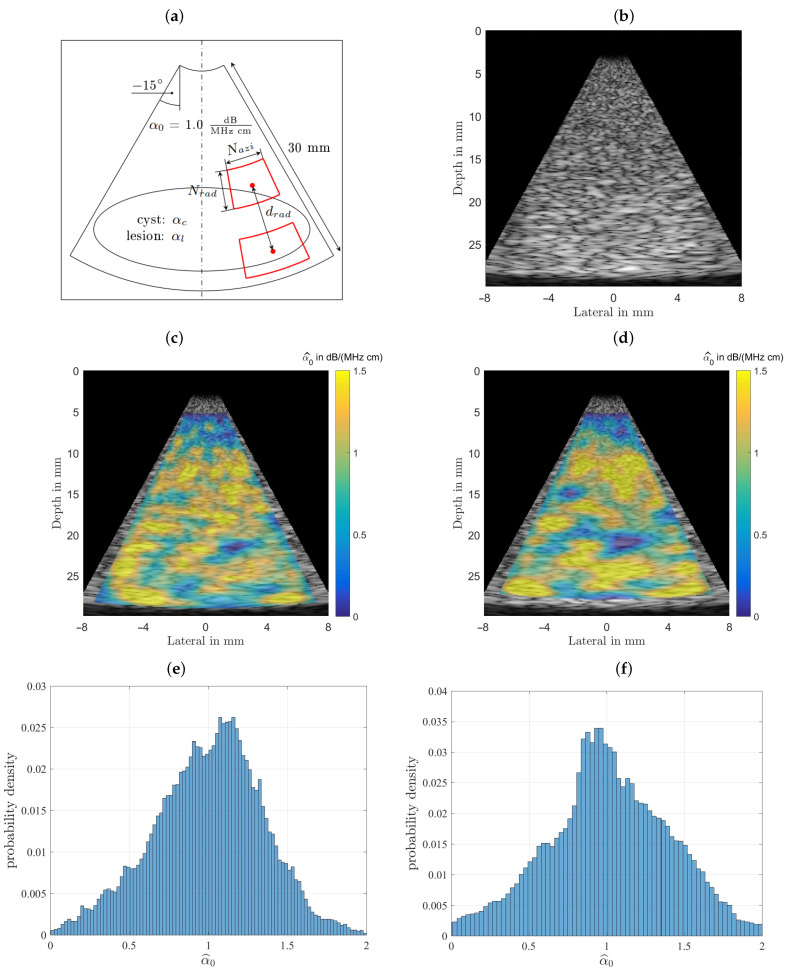
Comparison of the performance of both estimators. (**a**) The imaging geometry and the spectral density analysis windows. As a steering angle of $\pm 15^{\circ }$ would appear very slim, the image was stretched to square, leading to speckles that appear wider than those seen in real ultrasound images. Furthermore, the distal and proximal windows are displayed larger than they actually are, to give an idea of what is done here, meaning there is not as much averaging done than could be assumed by inspecting the graphic. (**b**) Shows the simulated B-mode image rendered with correct time-gain-compensation. This raw data is analyzed by both attenuation estimating algorithms. (**c**) The color-coded overlaid estimation result for the spectral-log-difference algorithm. (**d**) Estimation result for the method-of-moments. (**e**) Probability density function for the spectral-log-difference algorithm. (**f**) Probability density function for the method-of-moments.

Initially this phantom was designed and analyzed to characterize the estimator performance. For a fair comparison all B-mode scans were analyzed with equal settings for the power spectral estimator’s analysis window sizes and their respective depth for all subsequently characterized phantoms, with typical sizes as recommended in the literature [22,23]. The homogeneous phantom’s attenuation coefficient and the background of the other two phantoms were selected to lie above any value for benign breast tissue (fat, glandular tissue, etc.) at 1.0 dB/(MHz cm). For the homogeneous phantom both estimator’s performances are displayed in Figure 7 for visual comparison. Figure 7 shows the results obtained for the homogeneous phantom and by analyzing the middle row one can observe that both algorithms give visually similar results. The spread of the estimation can be seen in the bottom row, where the probability density functions, the histgrams are shown. In the left column the spectral-log-difference algorithm’s and in the right column results obtained for the MoM are shown. The spatially averaged mean over the complete sector scan gives ${\hat{\alpha }}_{0}{|}_{SLD}=0.9985$ dB/(MHz cm), for a true value $\alpha_{0}=1.00$. The standard deviation, obtained by again analyzing the complete sector scan was determined to be $\hat{\sigma }{\left(\alpha_{0}\right)}_{|SLD}=0.3245$ dB/(MHz cm).

The method-of-moments gives an ${\hat{\alpha }}_{0}{|}_{MoM}=1.0169$ dB/(MHz cm). The standard deviation, obtained by again analyzing the complete sector scan was determined to be $\hat{\sigma }{\left(\alpha_{0}\right)}_{|MoM}=0.3785$ dB/(MHz cm). The strength of the MoM, however, lies in the fact, that in addition to facilitating an estimate of the linear attenuation coefficient, it is also possible to estimate the power law exponent *y*, thus providing additional diagnostic cues.

**Figure 8:**
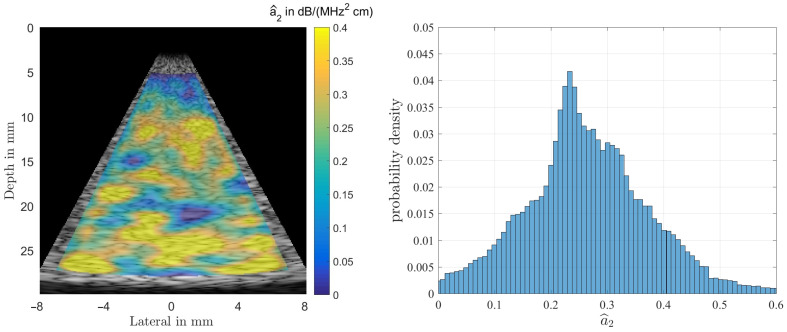
Result of estimating ${\hat{a}}_{2}$ from simulations of the homogeneous phantom (**left**). The true value is $a_{2}=0.2$, and the histogram for ${\hat{a}}_{2}$ (**right**). One can observe a rather large spread thus rendering the estimate to be of limited use in diagnosis.

Figure 8 shows the result of estimating the coefficient ${\hat{a}}_{2}$ from Equation (6) for the homogeneous phantom. As can be observed the overall structure of the color-coded estimation result follows the results displayed for $\alpha_{0}$ in Figure 7. This resemblance is probably due to the fact that all presented results were generated using the same randomly structured medium for all simulation runs. The spatially averaged mean over the complete sector scan gives ${\hat{a}}_{2}{|}_{MoM}=0.2648$ dB/(MHz${}^{2}$ cm), for the true value $a_{2}=0.20$. The standard deviation, obtained by again analyzing the complete sector scan was determined to be $\hat{\sigma }{\left(a_{2}\right)}_{|MoM}=0.1042$ dB/(MHz${}^{2}$ cm).

### 3.2. Cyst and Lesion Phantoms

The following results presented are for simulated phantoms designed to mimic either a cyst (with an attenuation coefficient close to that of water $\alpha_{0}=0.2$ dB/(MHz cm)), or a dense and highly attenuating lesion ($\alpha_{0}=2.5$ dB/(MHz cm)) located in a simulated breast tissue ($\alpha_{0}=1.0$ dB/(MHz cm)). Again, the power spectral density necessary as intermediate result is averaged over a rather small (approximately over an area of $N_{azi}\times N_{rad}\approx 1.30\times 1.35$ mm${}^{2}$) analysis window sized for sufficient spatial resolution to detect small structures.

**Figure 9:**
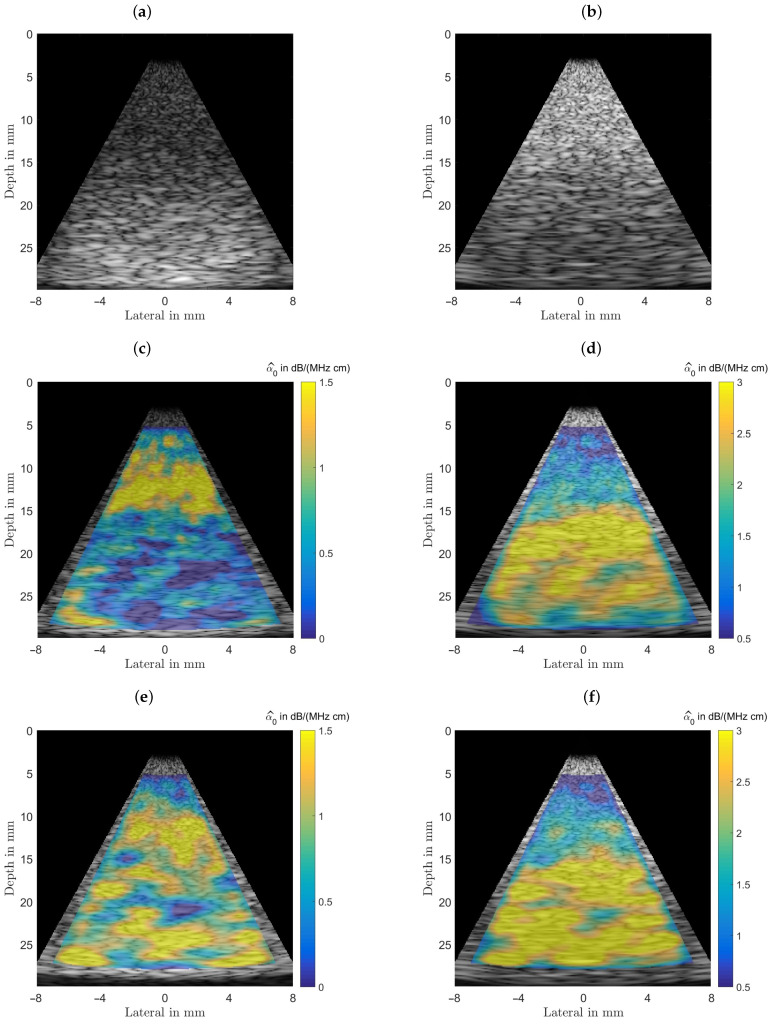
(**a**) B-mode images of an inclusion with a radius of 7.5 mm positioned centered at a depth of 20 mm, left, the inclusion is mimicking a cyst ($\alpha_{0}=0.2$ dB/(MHz cm)). (**b**) B-mode images of an inclusion with a radius of 7.5 mm positioned centered at a depth of 20 mm, the inclusion is mimicking a malignant lesion ($\alpha_{0}=2.5$ dB/(MHz cm)). (**c**,**d**) Estimation results obtained from the application of the spectral-log-difference algorithm. (**e**,**f**) Estimation results obtained from the method-of-moments.

The top row in Figure 9 shows the two B-mode images obtained from the simulated cyst phantom (left) and the lesion phantom (right) TGC-corrected for 1.0 dB/(MHz cm). In the middle row the resultant estimates are displayed color-coded via the spectral-log-difference method of the local attenuation coefficients. As can be seen from Table 3 the background attenuation is 1.00 dB/(MHz cm), the cyst and the lesion both with radii of 7.5 mm located centrally and at depths of 20 mm have attenuations of 0.2 dB/(MHz cm) and 2.5 dB/(MHz cm), respectively. It can be observed that the variance of the estimates for the structured media are visually similar to the homogeneous case. In the bottom row the resultant estimates via the method-of-moments of the local attenuation coefficients are displayed color-coded. Although, again visually appearing similar, the method-of-moments resulted in a larger variance.

### 3.3. Analysis of the Estimator’s Performance

Table 4 allows to compare both algorithms’ performances. The SLD-algorithm provides better results than the MoM in estimating $\alpha_{0}$ as indicated by the estimators’ standard deviation and is thus to be preferred over the MoM. If one were also interested in the power law exponent *y* as another cue on tissue type, the MoM is the only algorithm to also provide this information, albeit with a very large variance, that renders this capability rather useless.

In calculating the listed parameters for the cyst and the lesion numerical phantom the evaluated area was restricted to a rectangular shape lying completely within these structures (depth d=15 to 25 mm and azimuth covering $\pm 7.5^{\circ }$ only). Thus, a larger parameter variance is to be expected, as compared to the homogeneous case.

## 4. Discussion

As statistics show that one in eight women will develop invasive breast cancer in their lifetime and the key for a full recovery is early detection, it is of significant interest, to find a screening method that is cost effective, fast, risk-free and of highest accuracy, detecting even the onset of small lesions. This work was therefore initiated to help in the improvement of the diagnostic accuracy of breast cancer using noninvasive ultrasonic imaging.

Improvements are expected, if in addition to presenting an ultrasound image, an overlay of (color-coded) estimates of the acoustic properties of the different tissue types is possible. Since it is well documented that the attenuation coefficient significantly diverges between normal tissue and malignant lesions, we focused on the estimation of this parameter.

In our contribution we compared estimation results from two algorithms, the spectral-log-difference method and the method-of-moments and could show that, although the method-of-moments is able to provide the power law exponent as a second parameter for tissue characterization the spectral-log-difference method seems to provide results with lower estimation variance.

To verify that these methods are providing unbiased estimates with a variance close to the Cramér-Rao Lower Bound (CRLB), the estimator had to be applied to known properties and the results needed to be compared with measured values. Therefore part of this work was the investigation of different tissue-mimicking materials, for the production of tissue-mimicking phantoms with a set of desired acoustic properties and the investigation of suitable simulation software. Giving an insight of the *k*-Wave simulation environment, that, although requiring a high power computing environment, is well suited for 3D ultrasound simulations required in medical imaging.

As further steps in the research of this topic a closer analysis of the algorithms and the transition to data, i.e., to real US scanners, are scheduled.

## 5. Conclusions

Estimating tissue attenuation from ultrasonic B-mode scans especially if greater isonified depths need to be covered is a very involved problem resulting in a comparibly large estimator’s variance which to a good part is due to the unavoidable and also information conveying ultrasonic speckles. Since comparing two algorithms that have a very different mathematical basis and still seem to result in numerically very similar estimates hint on the cause of the variance not so much being due to the estimator but rather on the statistical properties of the analyzed medium.

We conclude that both algorithms deliver diagnostic cues for strongly deviating from normal tissue attenuation and both allow for a spatial resolution on the order of a few millimeters in azimuthal and radial directions.

## Figures and Tables

**Table 1:** Physical properties of different biological tissues, where ρ, is the mass density, *c*, the speed of sound and *Z*, the acoustic impedance, $\alpha_{0}$, the power law prefactor, *y*, the power law exponent, and B/A, the nonlinearity parameter [4] (except for lesion and cyst).

	ρ	*c*	*Z*	$\alpha_{0}$	*y*	B/A
Tissue	in kg m${}^{-3}$	in m s${}^{-1}$	in kg m${}^{-2}$ s${}^{-1}$	in dB/(MHz${}^{y}$ cm)	–	–
Blood	1060	1584	$1.679\cdot 10^{6}$	0.14	1.21	6
Bone	1990	3198	$6.364\cdot 10^{6}$	3.54	0.9	–
Breast	1020	1510	$1.540\cdot 10^{6}$	0.75	1.5	9.63
Fat	928	1430	$1.327\cdot 10^{6}$	0.6	1.0	10.3
Muscle	1041	1580	$1.645\cdot 10^{6}$	0.57	1.0	7.43
Lesion [12]	–	1549	–	1.28	–	–
Cyst [12]	–	1569	–	0.152	–	–
Water @ 20 ${}^{\circ }$C	1000	1482.3	$1.482\cdot 10^{6}$	0.002	2.0	4.96

**Table 2:** Weight composition of the IEC TMM (in % per weight) according to [16] (left) and the adjusted composition (right), which we used in the course of our research, dividing the proportions of glycerol and benzalkonium among the rest [15].

Component	Weight Component	Weight Component
	(%) Original	(%) Adjusted
Distilled, degassed, deionised water	82.97	93.9
Glycerol	11.21	0
Benzalkonium chloride	0.46	0
Agar	3.0	3.4
Silicon carbide (17 m)	0.53	0.6
Aluminium oxide (3.0 m)	0.95	1.1
Aluminium oxide (0.3 m)	0.88	1.0

**Table 3:** Parameters of the *k*-Wave simulations.

Phantom, Simulation, and	Homogeneous	Cyst	Lesion
Estimation Parameters	Phantom	Phantom	Phantom
grid spacing	30×30×30μm${}^{3}$	30×30×30μm${}^{3}$	30×30×30μm${}^{3}$
grid points	994×482×28	994×482×28	994×482×28
time step in ns	5.844	5.844	5.844
speed of sound *c* in m/s	1540	1540	1540
mass density ρ in kg/m${}^{3}$	1000	1000	1000
$\alpha_{0}$ in dB/(MHz cm)	1.00	1.00	1.00
$\alpha_{0}$ inclusion in dB/(MHz cm)	–	0.2	2.5
α-power	1.20	1.05	1.05
depth of inclusion in mm	–	20	20
radius of inclusion in mm	–	7.5	7.5
steering angle range	$\pm 15^{\circ }$	$\pm 15^{\circ }$	$\pm 15^{\circ }$
number of lines-of-sight (LOS)	201	201	201
transducer excitation frequency $f_{c}$ in MHz	5.0	5.0	5.0
transducer’s relative bandwidth	0.7	0.7	0.7
focal distance (azimuth) $F_{a}$ in mm	25	25	25
focal distance (elevation) $F_{e}$ in mm	400	400	400
$N_{azi}$ in LOS/in mrad/in mm @ *d* = 20 mm	25/65/1.30	25/65/1.30	25/65/1.30
$N_{rad}$ in mm	1.35	1.35	1.35
$\Delta d=d_{d}-d_{p}$ in mm	4.50	4.50	4.50

**Table 4:** Estimation results for all analyzed numerical phantoms.

	Homogeneous			Cyst			Lesion		
	Phantom			Phantom			Phantom		
	true	SLD	MoM	true	SLD	MoM	true	SLD	MoM
$\hat{\alpha_{0}}$	1.00	0.9985	1.0169	0.20	0.3116	0.2575	2.50	2.5368	2.8363
$\hat{\sigma }\left(\alpha_{0}\right)$	0.00	0.3245	0.3785	0.00	0.3405	0.4390	0.00	0.5634	0.6151
$\hat{a_{2}}$	0.20	n.a.	0.2648	0.05	n.a.	0.0732	0.05	n.a.	0.8904
$\hat{\sigma }\left(a_{2}\right)$	0.00	n.a.	0.1042	0.00	n.a.	0.1239	0.00	n.a.	0.2067

## Data Availability

Not applicable.
